# Tumor collagen framework from bright-field histology images predicts overall survival of breast carcinoma patients

**DOI:** 10.1038/s41598-021-94862-6

**Published:** 2021-07-29

**Authors:** Mindaugas Morkunas, Dovile Zilenaite, Aida Laurinaviciene, Povilas Treigys, Arvydas Laurinavicius

**Affiliations:** 1 Institute of Data Science and Digital Technologies, Vilnius University, Akademijos Str. 4, 08412 Vilnius, Lithuania; 2 National Center of Pathology, Affiliate of Vilnius University Hospital Santaros Klinikos, P. Baublio Str. 5, Vilnius, Lithuania; 3 Department of Pathology, Forensic Medicine and Pharmacology, Faculty of Medicine, Institute of Biomedical Sciences, Vilnius University, M. K. Ciurlionio Str. 21/27, 03101 Vilnius, Lithuania

**Keywords:** Breast cancer, Cancer imaging

## Abstract

Within the tumor microenvironment, specifically aligned collagen has been shown to stimulate tumor progression by directing the migration of metastatic cells along its structural framework. Tumor-associated collagen signatures (TACS) have been linked to breast cancer patient outcome. Robust and affordable methods for assessing biological information contained in collagen architecture need to be developed. We have developed a novel artificial neural network (ANN) based approach for tumor collagen segmentation from bright-field histology images and have tested it on a set of tissue microarray sections from early hormone receptor-positive invasive ductal breast carcinoma stained with Sirius Red (1 core per patient, n = 92). We designed and trained ANNs on sets of differently annotated image patches to segment collagen fibers and extracted 37 features of collagen fiber morphometry, density, orientation, texture, and fractal characteristics in the entire cohort. Independent instances of ANN models trained on highly differing annotations produced reasonably concordant collagen segmentation masks and allowed reliable prognostic Cox regression models (with likelihood ratios 14.11–22.99, at *p*-value < 0.05) superior to conventional clinical parameters (size of the primary tumor (T), regional lymph node status (N), histological grade (G), and patient age). Additionally, we noted statistically significant differences of collagen features between tumor grade groups, and the factor analysis revealed features resembling the TACS concept. Our proposed method offers collagen framework segmentation from bright-field histology images and provides novel image-based features for better breast cancer patient prognostication.

Collagen is a major structural component of the extracellular matrix (ECM); its fibers connect to form a supportive environment for growing cells and tissues and thus have an important role in tumorigenesis. Collagen abundance correlates with high mammographic density (HMD) that, in turn, is an independent risk factor for developing breast cancer (BC)^1–4^. Moreover, in women free of invasive or non-invasive neoplastic lesions but with the high BC risk profile, altered stromal collagen organization was observed in HMD breast tissue^3,4^. In breast tumors, collagen-dense microenvironment may have multiple impacts: it can be viewed as a static, space-filling material in which tumor cells are embedded, also known to stimulate metastatic tumor progression by directing the migration of malignant cells along the straightened and aligned structure of ECM towards the blood vessels^5,6^. In addition to this “biomechanical” aspect, collagen also participates in biological modulation of cellular events by interacting with specific cellular receptors to trigger various signaling pathways. Moreover, biochemical and biomechanical properties of the collagen-rich ECM network facilitate a barrier formation and alter drug-diffusion through the tumor tissue, thus adding another complexity layer to the collagen framework^7,8^.

While aspects of collagen framework structural changes across different cancer types prove their prognostic value^9,10^, collagen imaging techniques are also being developed. Even though the collagen-rich stroma of the tumor tissue is clearly distinguishable in ordinary haematoxylin and eosin (H&E) staining, and also some well known and routinely used collagen-specific histochemical stains provide a more detailed picture^11^, a significant amount of effort is being put to develop specialized imaging modalities allowing exact detection of collagen fibers in pathology samples. Birefringent collagen fibers can be visualized under polarized light^12^, additional staining with Sirius Red (SR) acts as an intensifier of natural collagen birefringence since elongated dye molecules align with the collagen fibers, making this combination a prominent technology for collagen structural analysis^13,14^. By exploiting the hyperpolarizability property of collagen molecular structure, the second harmonic generation (SHG) microscopy has become a solid tool that can be applied to label-free specimens of many collagen-related pathological conditions. The most prominent use of SHG microscopy in collagen studies was to produce tumor-associated collagen signatures and associate them with patient prognosis^15,16^. Progressing breast tumors pass a series of evolutionary stages that can be characterized by specific collagen organization: an early stage of increased collagen deposition near the lesion site (TACS-1), a stage of advancing growth with straight collagen fibers aligned to constrain the tumor volume (TACS-2), and a stage of invasion and spread along collagen fibers aligned perpendicularly to the tumor boundary (TACS-3).

A spectral phasors approach applied to multispectral fluorescence images of H&E stained tissue slides enables straightforward collagen segmentation^17^. A collagen-specific signal cluster in the phasor space can be identified and mapped to the corresponding H&E image resulting in a synthetic image mimicking Masson trichrome staining and is even more precise than SHG or polarized light microscopy (PLM) since it also captures non-birefringent collagen. Image-based collagen biomarkers and the potential clinical value of this technique remain to be explored.

Although both SHG and PLM, as well as some other techniques, have been used to image collagen at high resolution, the use of the specialized imaging modalities is generally limited to the research (due to the relatively high cost of equipment and lack of whole slide imaging capacity). Meanwhile, more accessible bright-field microscopy methods cannot offer high precision, even though there are few approaches like manual thresholding of hue, brightness, and saturation, or stain separation using color deconvolution^18,19^. However, these collagen detecting and segmenting methods are sensitive to day-to-day laboratory variation of the staining quality. To overcome this type of variation, previous studies employed measurement of collagen fiber angles by hand and showed that collagen organization could be adequately measured by human observers and associated with the response to chemotherapy^20^.

Few studies explore the feasibility of neural networks to detect and segment tissue collagen in bright-field microscopy images. Jung et al.^21^ presented deep convolutional neural networks applied to tissue collagen detection. Graph analytics was applied to collagen deposits segmented by a neural network from histopathology images of simian immunodeficiency virus-infected rhesus monkeys to detect collagen morphological changes in the course of infection. In a more recent study, Keikhosravi et al.^22^ proposed the deep convolutional neural network-driven bright-field H&E to SHG image transformation and were able to produce synthetic SHG-like images of remarkably high detail level. These studies demonstrate the potential of deep convolutional neural networks for collagen architecture assessment, although clinically valid image-based collagen framework indicators remain to be developed and tested.

In this study, we trained an ANN to segment the collagen framework in bright-field microscopy images of BC tissue microarray. We explore the impact of different ANN training modes on collagen predictions by intersection, coverage, and ratio analysis of generated collagen segmentation mask. We demonstrate the prognostic value of the quantitative indicators based on the shape, orientation, and texture features of the collagen fiber framework. Significant associations between computed collagen features and tumor growth patterns were noted.

## Materials and methods

### Patients and tissue methods

Two hundred three patients involved in this and our previous studies^23,24^ underwent surgery during 2007–2009 at the National Cancer Institute (Lithuania, Vilnius). During this period, tumor samples were collected prospectively, and the pathologist’s examination of these samples was performed at the National Centre of Pathology (Lithuania, Vilnius). 107 patients were diagnosed with an early-stage hormone receptor-positive invasive ductal breast carcinoma. Tumor tissue samples from 92 patients were used for the analyses. Informed written consent was obtained from all patients participated in the study. All methods were carried out in accordance with relevant guidelines and regulations and the study was approved by the Lithuanian Bioethics Committee (reference number: 40, date 2007-04-26, updated 2017-09-12). Clinicopathological characteristics and follow-up data from these patients are reported as the mean and median values for continuous variables and the frequencies of categorical variables and are given in Table 1.

**Table 1:** Characteristics of patients with hormone receptor positive breast carcinoma.

Clinicopathological parameters	Patients (%)
Total	92 (100%)
**Outcomes**	
Survived	72 (78.26%)
Died	20 (21.74%)
**Age**, **years**	
Mean	57.27
Median	59
Range	27–87
**Gender**	
Female	92 (100%)
Male	0 (0%)
**Histological grade (G)**	
G1	19 (20.65%)
G2	44 (47.83%)
G3	29 (31.52%)
**Tumour invasion stage (T)**	
T1	52 (56.52%)
T2	40 (43.48%)
T3	0 (0%)
T4	0 (0%)
**Lymph node metastasis status (N)**	
N0	48 (51.17%)
N1	30 (32.61%)
N2	11 (11.83%)
N3	3 (3.26%)
**Intrinsic subtype**	
Luminal A	49 (53.26%)
Luminal B, HER2 negative	26 (28.26%)
Luminal B, HER2 possitive	17 (18.48%)

Patients in this group were females at the age of 27 to 87 years who have been followed for a period of 17 to 121 months after surgery. Hormone receptor positivity was defined previously^24^ as an estrogen receptor or progesterone receptor immunohistochemical (IHC) positivity in at least 1% of tumor cells.

Tissue microarray (TMA) samples (1 mm diameter spot per patient) randomly selected in intratumoral regions were used for the study. Ki67 IHC slides were additionally stained with 0.1% Sirius Red in Picric acid. The slides were scanned using the Aperio ScanScope XT Slide Scanner at 20× objective magnification (0.5 µm per pixel). Images of single TMA cores were extracted from whole-slide images for further analysis.

### Generation of ground truth for collagen segmentation

**Figure 1:**
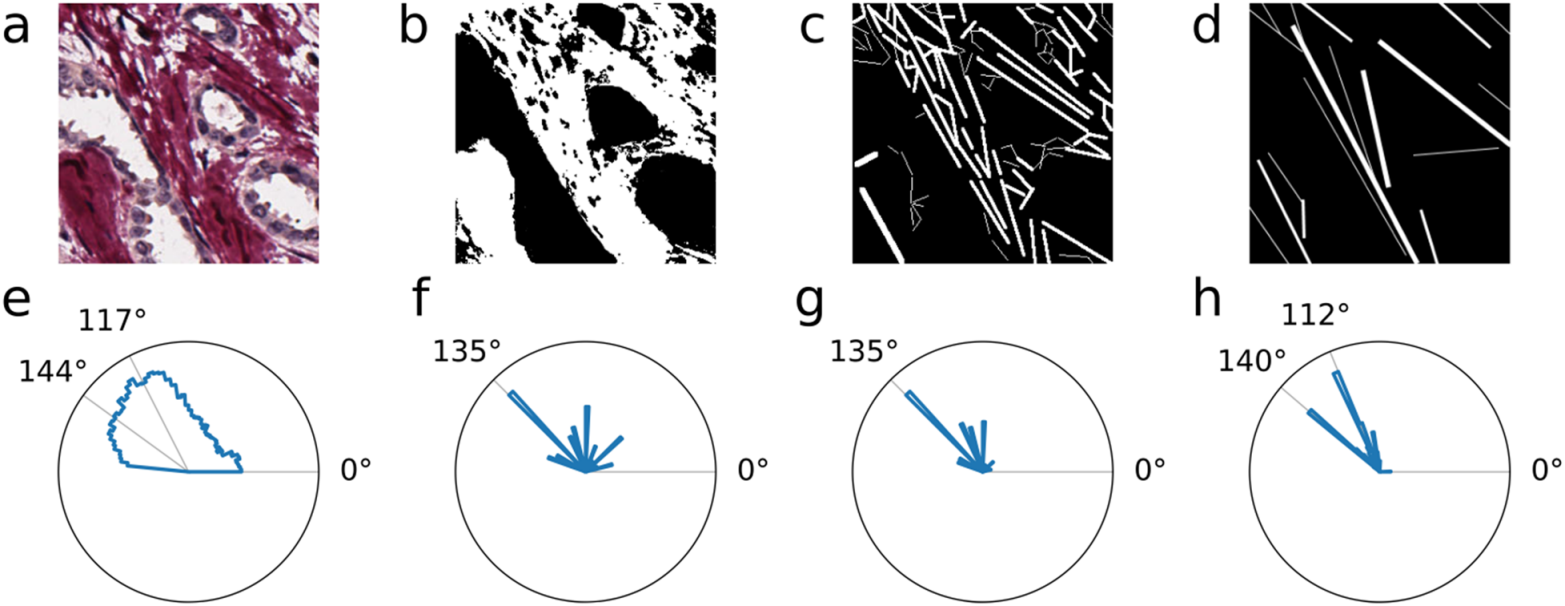
Annotation consistency. (**a**) SR stained breast carcinoma tissue. (**b**) binary annotation mask produced by semi-automated method. (**c** and **d**) binary annotation masks produced manually. (**e**–**h**) polar projections of histograms of orientations captured by HOG procedure from corresponding (**a**–**d**) images. Note that all annotations differ by the level of detail but considerably agree on orientation. Thin annotation lines in binary images may apear as gray because of downsizing. For more examples of annotations please see Supplementary Fig. S1.

Initial dataset intended to train the model contained 116 original image patches of 256 × 256 pixels size that were randomly cropped from 48 TMA core images and manually annotated. Two professionals (MM and DZ), blinded to each other, were asked to give a rough estimation of the collagen framework motifs during the annotation process. Experts could place a set of straight lines of varying thickness on parts of image patches to capture the direction of collagen fibers. As an alternative, the third set of annotations was generated by image thresholding, followed by manual curation (see Fig. 1 and Supplementary Fig. S1). Augmentation transformations to annotated images, including horizontal and vertical flips, rotations by 90, 180, 270 degrees, were applied to expand the training image dataset to 696 patches. Before training the segmentation models, we applied different morphological image dilation amounts to ground truth masks using a 5 × 5 elliptic structuring element (number of iterations—nits = 0, 1, 2, 3, see Supplementary Fig. S3 and Supplementary Table S2).

### U-net neural network for collagen framework segmentation

The ANN employed in this study is a fully convolutional encoder-decoder network (named “U-net”) developed for biomedical image segmentation^25^. This network architecture supports pixel-level localization of detected objects by concatenating compact encoded feature maps with corresponding sparse decoded features at multiple scales inside the network’s hidden layers. We modified the original U-net architecture to accept input images of 256 × 256 pixels size. In our convolutional layers, we substitute rectified linear units for exponential linear units and use the input’s padding to ensure that the output has the same shape as the original input. We composed the network of 64 convolutional layers, including five transposed convolutions in the up-sampling path. The last convolutional layer (output layer) maps the feature space of the final layer on the up-sampling path to a single class probability image representation via 1 × 1 convolutions followed by a sigmoid activation function (for the detailed architecture of an ANN see Supplementary Fig. S2).

In this study, we introduced an additional block to the original U-net architecture. The block receives a tensor from the previous layer and passes it down the computational graph in two parallel flows, each composed of three 2D convolutional layers and one dropout layer. In its first internal layer, one flow has a single channel 2D convolution—a bottle-neck, and the parallel flow has a multichannel 2D convolution—an expansion layer. Output tensors from both flows have an identical shape; thus, after concatenation, they contribute equally to the network in terms of feature maps. We put these “bottle-neck” blocks on the network’s encoder path, after each max-pooling layer.

To minimize the binary cross-entropy loss function, we trained the network with adaptive moment estimation using default parameters provided in the original method^26^. We trained the model on single patch batches and randomly split the dataset of annotated patches into the training subset (80%) and validation subset (20%). We set the algorithm to save model weights after each improvement in validation loss, and terminate the training phase after validation loss did not improve for 20 consecutive epochs. We expected the ANN to learn the representation of human visual perception.

### Principle of collagen framework detection by neural network

**Figure 2:**
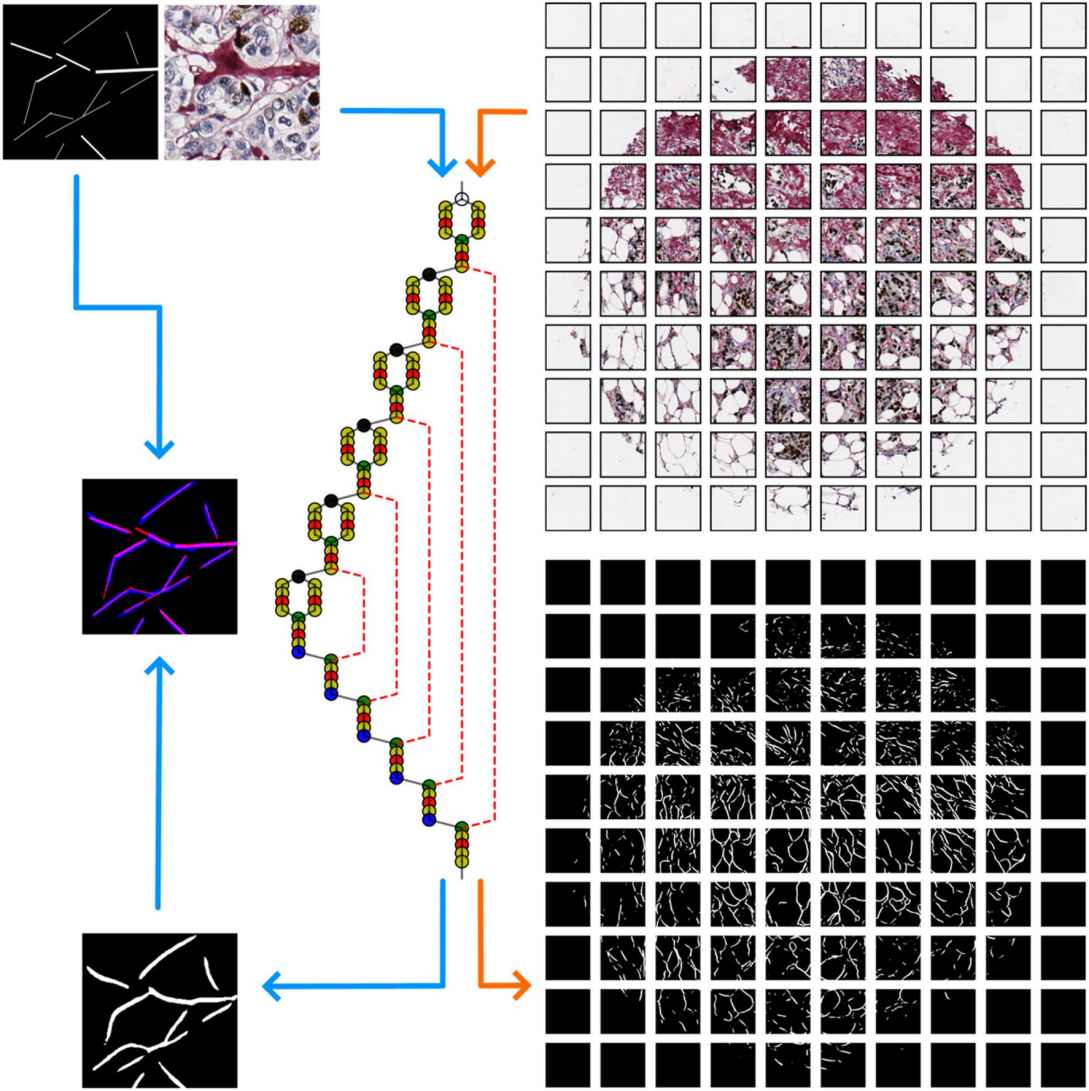
Principal workflow design. The ANN (in the middle) is trained on annotated image patches (on the left). Training is guided by the binary cross-entropy loss, and is evaluated by mean IoU. Training phase is indicated by blue arrows. After the training phase is over, the ANN accepts new images (on the right) and produces collagen segmentation masks (CSMs). Testing phase is indicated by orange arrows. Detailed ANN architecture is given in Supplementary Fig. S2.

An overview of the workflow is given in Fig. 2. To fit the model input shape, we split the target image into overlapping (128-pixel step-size in vertical and horizontal directions on an image plane) 256 × 256 pixel size patches. The trained model performs predictions patch-by-patch to produce probability maps that, in turn, are subjected to thresholding. Each pixel in a probability map receives a value of 1.0 if a probability of detecting collagen in that pixel is higher than 0.5. Otherwise, the pixel receives a value of 0.0. To avoid prediction artifacts at patch borders, we consider correct predictions to be present in both overlapping patch-level probability maps. We merge patch-level results to form a binary collagen segmentation mask (CSM) of an original image of a single TMA spot. Lastly, we apply the size filtering of detected objects removing objects containing less than 50 pixels.

### Model evaluation

During the training phase, model progress was monitored by prediction-annotation similarity. We employed an intersection over union (IoU) metrics:

$$IoU = \frac{TP}{{FP + TP + FN}}$$

where TP, FP, and FN are true positive, false positive, and false negative pixels. For pairwise comparison of ANN models at a single CSM level, the similarity was aggregated as a mean ratio of non-empty pixel counts between CSMs, mean coverage, and mean IoU of all analyzed CSMs:

$$\begin{aligned} mean\_ratio & = \frac{1}{N}\mathop \sum \limits_{i = 1}^{N} \frac{{CSM_{i,mod1} }}{{CSM_{i,mod2} }} \\ mean\_coverage & = \frac{1}{N}\mathop \sum \limits_{i = 1}^{N} \frac{{CSM_{i,mod1} \cap CSM_{i,mod2} }}{{CSM_{i,mod1} }} \\ mean\_IoU & = \frac{1}{N}\mathop \sum \limits_{i = 1}^{N} \frac{{CSM_{i,mod1} \cap CSM_{i,mod2} }}{{CSM_{i,mod1} \cup CSM_{i,mod2} }} \\ \end{aligned}$$

where N is the number of images compared, and *mod* is an ANN model. Since no ground truth was available at a full image level, we greatly relied on visual estimation of consensual information in segmentation masks resulting from different models.

### Quantitative feature extraction

We analyzed the collagen framework by computing 37 multi-level features that fall into three major groups: pixel-level features such as angle and magnitude of the orientation of edges present in an area surrounding a pixel in the CSM; fiber-level features include morphometric measures of each detected fiber; image-level features such as fractal characteristics and texture descriptors of the pixel-level feature representation images.

Since the study used a single TMA core image per patient, all image-level features also represent the patient-level. Fiber-level and pixel-level features were extracted and aggregated into patient-level by the mean, median, or standard deviation.

### Pixel-level features

Fiber orientation features for each pixel in the target image were measured. We have empirically selected an 18 × 18 pixels size bounding box (context area) for a pixel of interest. Unsigned gradients (originating from edges present in an image) were summarized in 64 evenly spread histogram bins covering a range of 0–180 degree angles. We have used a Sobel operator^27^ in both x and y directions in an image plane to estimate gradients present in an image. Histogram of oriented gradients (HOG) procedure then counts occurrences of gradient orientation in image patches and assigns the gradient magnitude of each pixel to the corresponding histogram bin covering a particular angle. We could summarize orientation angle descriptors of the collagen framework by the linear directional mean, circular variance, and circular standard deviation from the histogram. Since the fiber orientation angle is dependent upon tissue placement on the glass slide, the only meaningful fiber orientation angle related feature at the patient-level was the circular standard deviation (CSD, see Table 2). In contrast, we summarized the magnitude of gradients by the mean and standard deviation (for histogram values where magnitude was not zero).

**Table 2:** Feature list.

**Orientation**		
LDM	Linear directional mean	
CV	Circular variance	
CSD	Circular standard deviation	
mMag	Mean magnitude	
stdMag	Standard deviation of the magnitude	
**Morphometry**		
mFL	Length	Mean
mdFL		Median
stdFL		Standard deviation
mFP	Path	Mean
mdFP		Median
stdFP		Standard deviation
mFS	Straightness	Mean
mdFS		Median
stdFS		Standard deviation
mFW	Width	Nean
mdFW		Median
stdFW		Standard deviation
**Density**		
FD	Number of pixels in the mask	
nENDP	Number of endpoints	
mD	Distance between endpoints	Mean
mdD		Median
stdD		Standard deviation
**Texture**		
Energy		
Contrast		
Correlation		
Inertia		
Homogeneity		
Sum average		
Sum variance		
Sum entropy		
Entropy		
Difference variance		
Difference entropy		
Informational measure of correlation 1		
Informational measure of correlation 2		
**Fractal**		
Fractal dimension		
Lacunarity		

### Object-level features

We computed morphometric features of collagen fibers treating each fiber as a separate object and subsequently aggregated into patient-level features by mean values. Fiber objects were bound in a minimum bounding rectangle, and a diagonal of this rectangle was used as a fiber length (FL) measure. The length of a fiber centerline, or the fiber path (FP—defined as a line that divides a fiber into two equal parts along its longer axis), was calculated as half of the total number of points in a fiber contour. We computed the fiber width (FW) as half the Euclidean distance between all opposing pairs of points in the fiber contour aggregated by the median. Fiber straightness (FS) was calculated as a ratio of FL over FP. Fiber density (FD) was computed as a mean Euclidean distance from each detected fiber endpoint to all neighbor endpoints in a CSM.

### Image-level features

We computed fractal characteristics and texture features of the collagen framework from the CSMs. Box counting procedure^28^ was employed to compute the fractal dimension and the lacunarity. The spatial gray-level co-occurrence matrix calculated with a 1 pixel displacement vector was used to derive image texture descriptors^29^, including energy, contrast, homogeneity, and entropy.

### Statistical analysis

We evaluated the consistency of expert annotations by Bland–Altman difference analysis, one-sample t-test on differences against the zero value, and an independent sample t-test on cases in 95% agreement interval.

In an exploratory analysis, we used Shapiro–Wilk and Levene’s test for data normality and homogeneity assumptions. One-way ANOVA and Tukey’s HSD post hoc test were used to assess differences between group means.

We performed factor analysis with the principal component method using a covariance matrix of Pearson’s correlations of the variables. To simplify the structure of factors and improve the interpretation, we applied varimax rotation.

Cutoff points for variables were determined, and patients were stratified into groups based on statistical differences as assessed by the log-rank test. In univariate analysis we used Kaplan–Meier estimates to assess patient survival and performed feature validation following a leave-one-out strategy, as described previously^30^. To model the effect of multiple variables on patients’ survival time, we applied Cox proportional hazards (Cox regression) analysis.

For all tests to prove the significance, we accepted a *p*-value < 0.05.

### Implementation

For deep learning, we used TensorFlow^31^ framework in Python. Full-training (training from scratch) of the model was performed on a high-performance graphical processing unit (Nvidia GeForce GTX1080). Feature extraction and quantification were implemented using the “scikit-image” image processing library in Python^32^. All statistical analyses were performed in the R statistical environment. For univariate survival analysis, we used the “Cutoff Finder” algorithm^33^ and the “survival” package^34^ for multivariate statistical modeling.

### Ethics approval and consent to participate

The Lithuanian Bioethics Committee approved this study (Reference No.: 40, date 2007-04-26, updated 2017-09-12).

## Results

### Expert annotation consistency

Regions containing fibrous collagen can be appreciated visually in SR-stained or even in H&E bright-field microscopy images; also, the HOG procedure enables reliable detection of the fiber orientation (see Fig. 1b, d). We evaluated the consistency of the collagen framework annotation procedure by Bland–Altman difference analysis of the two “manual” approaches focusing on differences in the count of annotated objects, average object size (in pixels), and the dominant orientation of annotated objects (in degrees). The analysis revealed significant differences in the level of detail the experts put into their annotations, as well as the orientation of marked objects. The one-sample t-test on differences against the zero value shows the presence of fixed bias for all parameters evaluated (*p*-value < 0.05 for all estimates). However, in the 95% agreement interval, the differences in annotation orientations are not significant (*p*-value = 0.628, see Supplementary Table S1).

### Model prediction consistency

To explore the ability of ANNs to produce consistent segmentations, we have trained the modified U-net model on ground truth obtained from three sources (low detail manual, high detail manual, and semi-automated) to produce M1, M2, and M3 model instances. To further investigate the impact of annotation precision on collagen segmentations, we trained each of M1, M2, and M3 on enhanced ground truth masks by applying varying amounts of morphological dilation to the original annotations (see Supplementary Fig. S3). Training yielded 12 instances of independent models (see Supplementary Table S2). To evaluate the effect of different ground truth on segmentation accuracy, we performed pairwise comparison of CSMs obtained from all independently trained models. We analyzed the impact of dilation on model predictions by visual comparison as well as by ratio of areas, intersection, and coverage (see Supplementary Fig. S4). When raw annotations were used (nits = 0), models did not produce meaningful results (by visual assessment), except for M3. Annotation dilation did not affect M3 but substantially pushed M1 and M2 towards M3, and each other. By varying the amount of dilation, we could reach model agreement over 0.60 for M3 versus M2, 0.50 for M3 versus M1, and 0.40 for M2 versus M1 (as evaluated by mean IoU). With an increasing amount of dilation, CSMs from M2 even outgrow those from M3 by area (reaching a mean ratio of 1.17) but target different parts of test images because mean coverage drops below 80%. Mean coverage analysis quantifies the proportion of one CSM incorporated in the other. In this context, M1, to a great extent (over 95%), is included in both M2 and M3. While CSMs of M1 and M2 demonstrate considerable agreement even without annotation enhancement, CSMs of M3 are considerably larger by area (area ratios 0.01–0.04 for M1 vs. M3, and M2 vs. M3 at nits = 0). We could obtain better agreement for M1 vs. M3 and M2 vs. M3 with minimal morphological dilation (area ratios 0. 17–0.42, at nits = 1). Therefore, for further factor analysis and univariate and multivariate prognostic analyses, we have selected to compare the CSMs obtained by M1, M2, and M3 models trained on least enhanced annotations (nits = 1, see Supplementary Table S2).

### Visual motifs of the predicted collagen framework

**Figure 3:**
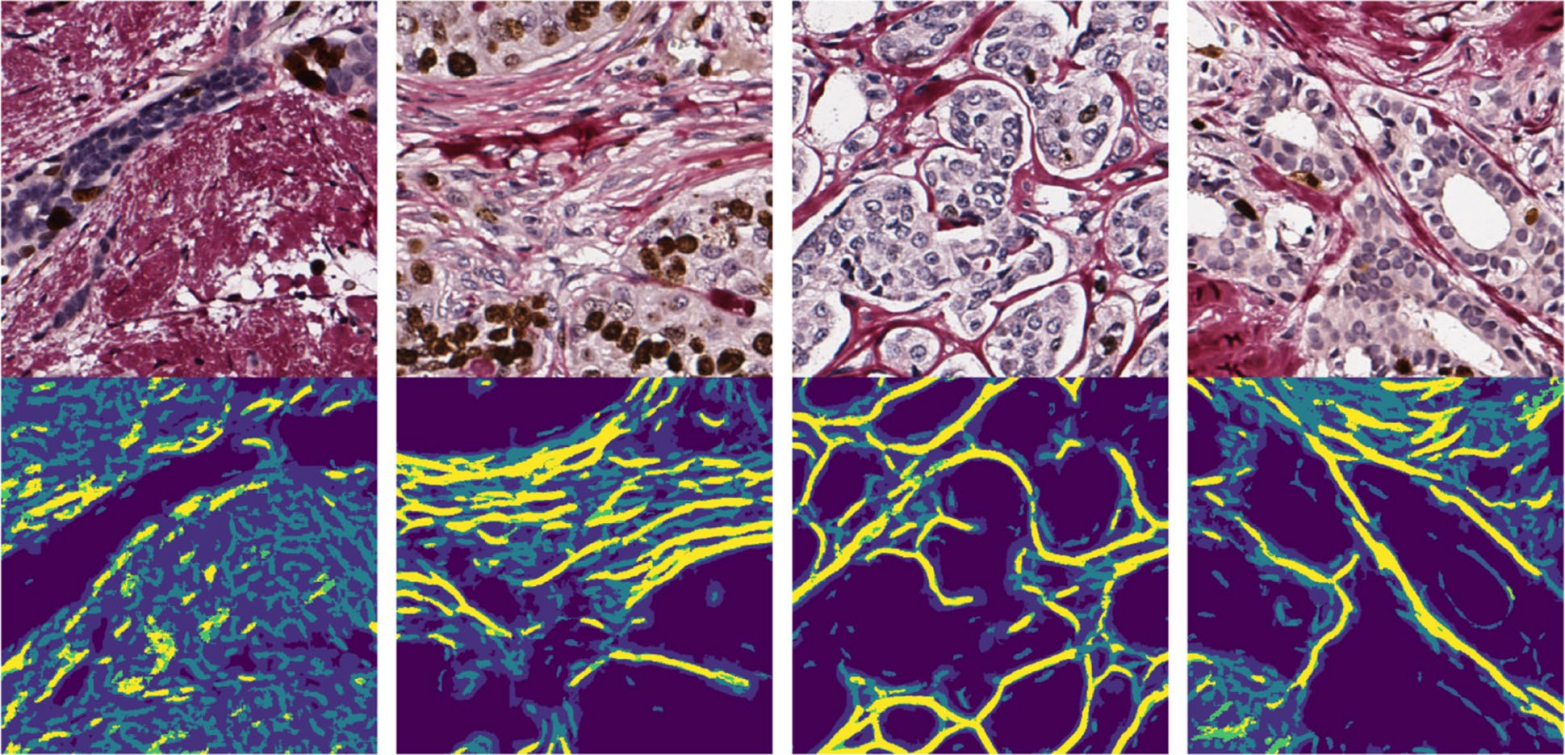
Examples of CSMs. Collagen framework segmentation masks (bottom row) extracted from Ki67-SR-stained BC TMA images (top row). In an overlay of CSMs from different ANN models bright yellow color indicates regions where all models agree, and darkest blue color indicates background. Lighter shades of blue indicate M2 and M3. Yellow-colored area covers over 80% of M1. For high resolution examples please reffer to Supplementary Fig. S5.

On the largest scale, the predicted collagen framework resembles a web-like structure (for high-resolution CSM examples see Supplementary Fig. S5). While CSMs from the M3 ANN model mostly capture bulk collagen, those from M1 and M2 are composed of disconnected structural elements that vary greatly in number and appearance. The smallest components of the framework (typically in CSMs of M1) arise from tissue image parts containing fragmented, fibrous stroma. Most often present in highly cellular tumor samples, these fiber-like objects are of simple geometry, scattered, and disconnected from the larger structures. Fibers spanning sparse intercellular space appear longer and tend to curve around cell islets. Longer fibers extend through regions of well-defined oriented collagen despite SR staining intensity. In CSMs from M1 and M2, intense staining often yields few, relatively short, and disordered branches. In contrast, fibrotic stroma regions yield notably longer fibers. In fibrosis, we can observe fibers branching, merging, and forming loops—these dense and more complex structures form fiber bundles—homogeneous motifs of long, parallel, interconnected branches, outlining large clusters of neoplastic cells (well represented in CSMs from all models, see Fig. 3). In most extreme cases, fibers bundle into complex textures, almost complete circles, mesh-like structures.

### Factor analysis of computed collagen framework features

To understand the general properties of collagen framework architecture, we analyzed three models trained with annotations by different experts and the least amount of processing applied (nits = 1 of morphological dilation, see Supplementary Table S2).

**Figure 4:**
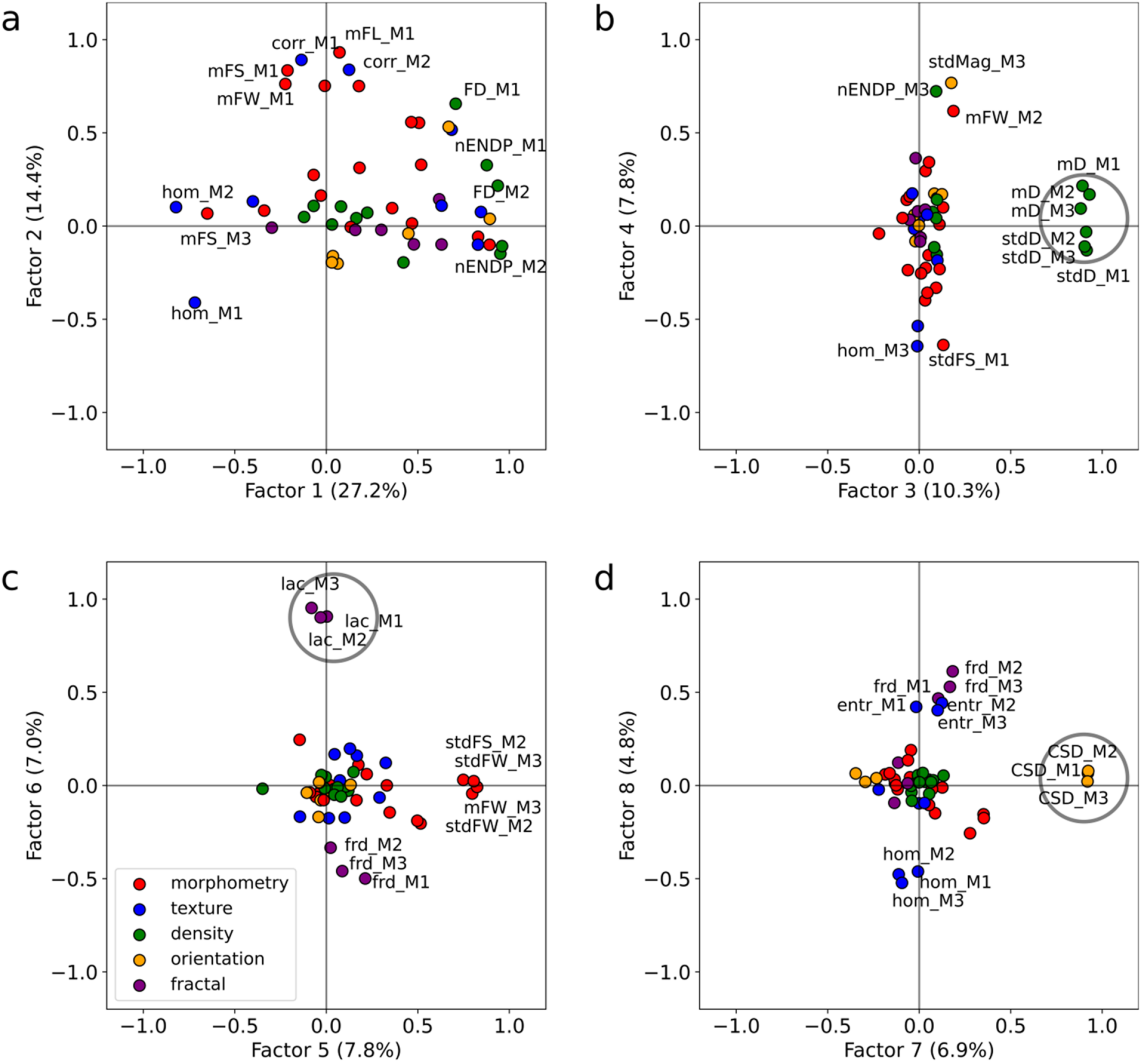
Rotated factor patterns. Factors 3, 6 and 7—density (**b**), lacunarity (**c**), and orientation (**d**) features from all ANN models aggregate in orthogonally independent factors (circled). Proportion of variance explained by the factor is given on axes next to corresponding factor names. In total, 8 factors explain 86.2% of variance in the data.

We selected a set of textural, morphometric, orientation, and density descriptors of CSMs and performed a factor analysis to uncover latent relationships governing collagen arrangement in hormone receptor-positive BC. Eight independent factors (with eigenvalues ≥ 1) explain 86.2% of the variance in the data. Corresponding patterns are visualized in Fig. 4. With rare exceptions, similar features from all three different models form independent factors. Strong loadings of density (FD, nENDP) and texture (image entropy) from all three models combine in Factor 1 (see Fig. 4a). The variance of orientation magnitude (stdMag) from M1 and M2 and the mean and variance of the fiber length (mFL, stdFL) from M3 also contribute to Factor 1. Factor 2 is mainly composed of the morphology features of M1 (FS, FW, FL). Densities measured as the mean and variance of the distance between fiber endpoints (mD, stdD) from all models form Factor 3 (see Fig. 4b). Density (nENDP) and the variance of orientation magnitude (stdMag) from M3 form Factor 4. The variance of the fiber width and straightness from M2 and M3 represent Factor 5. Factors 6, 7, and 8 are respectively composed of the lacunarity (lac), the variance of orientation angle (CSD), and fractal dimension (frd) from all three models (see Fig. 4c, d).

### Association of collagen features with tumor grade

The one-way ANOVA revealed statistically significant differences of means of collagen framework features between tumor grade groups (see Supplementary Table S3). In M1 CSMs, lower packaging dimension (frd↓) distinguished high-grade (G3) from low-grade (G1, G2) tumors. More homogenous (homogeneity↑), less densely arranged (FD↓, nENDP↓, frd↓) collagen framework, and less scattered fibers (stdMag↓) in M2 CSMs were more indicative of high-grade tumor. Higher fiber straightness (mFS↑) and lower fiber density (FD↓) in M3 CSMs were characteristic of high-grade tumors.

### Univariate and multivariate modeling of survival predictors

To predict patient survival, we selected candidate features in Kaplan–Meier univariate analysis by significantly (*p*-value < 0.05) enhanced or decreased hazard ratio (HR). Variables extracted from the CSMs of all ANN models (M1, M2, and M3) allowed prognostic dichotomization of the patients (see Table 3). Subsequently, in a leave-one-out cross-validation procedure (see Supplementary Table S4), we selected sets of highest-ranking features and used them together with conventional pathological-clinical indicators (T and N categories, tumor grade, patient age) in the Cox proportional-hazards modeling. Each resulting Cox regression model contained features of CSMs of different ANN models. At least one fiber morphometry feature was present in all models—mFW in M1 (HR = 14.25), mdFS in M2 (HR = 0.12), and stdFW in M3 (HR = 5.01). The variance of orientation magnitude (stdMag) appeared in two models (M1, HR = 2.69 and M3, HR = 4.07) and texture correlation once (in M2, HR = 4.54). In any scenario, no clinicopathological indicators entered the models (see Table 4 for multivariate prognostic model details, and Supplementary Fig. S6 for Kaplan–Meier plots of prognostic features obtained in univariate prognostic modeling).

**Table 3:** Univariate analysis.

Clinicopathological indicators		*p*-value	HR
T category (T1 vs. T2)		0.645	0.81
N category (N0 vs. N1-3)		0.200	1.79
Histological grade (G1-2 vs. G3)		1.000	1.00
Subtype (LumA vs. LumB-LumBHER2+)		0.630	1.24
Age (≤ 59 vs. > 59)		0.062	2.62

M1	Cutoff value	*p*-value	HR
mFW	5.238	< 0.001	17.75
mdFW	5.646	< 0.001	9.87
mFS	0.596	0.002	5.67
Correlation	0.858	0.012	5.31
mdFP	43.250	< 0.001	5.12
mdFS	0.610	0.001	4.66
mdFL	26.593	0.001	4.48
stdMag	58444.629	0.001	4.18
mFP	64.099	0.022	3.79
mFL	42.916	0.031	3.14
FD	446163.500	0.025	2.74
mMag	83522.157	0.026	2.62

M2	Cutoff value	*p*-value	HR
mFS	0.559	0.010	3.08
Correlation	0.850	0.024	2.7
Informational measure of correlation 1	− 0.678	0.015	0.35
mdFS	0.538	0.001	0.23

M3	Cutoff value	*p*-value	HR
sdFW	4.012	0.026	3.67
mFW	8.539	0.007	3.14
mdFW	8.902	0.010	3.06
stdMag	54368.033	0.011	2.98
sdD	517.382	0.019	2.86
mFP	883.345	0.031	2.74
mdD	1059.998	0.025	2.64
mD	1081.991	0.038	2.56
mdFS	0.514	0.031	0.35
CSD	0.766	0.004	0.27
CV	0.254	0.004	0.27

Clinicopathological indicators can not stratify early-stage hormone receptor-positive invasive ductal breast carcinoma patients into significant prognostic groups. Image features producing significant patient stratification are grouped by the ANN model they were generated. *LumA* Luminal A, *LumB* Luminal B HER2 negative, *LumBHER2*+ Luminal B HER2 positive.

**Table 4:** Multivariate Cox regression analysis.

	HR	*p*-value	95% confidence
**Model 1 (LR: 22.99, *p*-value = 1 × 10**^**−5**^**)**			
stdMag	2.69	0.029	1.11–6.55
mFW	14.25	0.010	1.88–108.20
**Model 2 (LR: 16.21, *p*-value = 3 × 10**^**−4**^**)**			
mdFS	0.12	< 0.001	0.04–0.37
correlation	4.54	0.003	1.65–12.49
**Model 3 (LR: 14.11, *p*-value = 9 × 10**^**−4**^**)**			
stdMag	4.07	0.002	1.66–9.97
stdFW	5.01	0.011	1.44–17.43

Each Cox regression model was obtained from the features of different ANN models and was named accordingly.

## Discussion

In this study, we explored the informative value of bright-field microscopy images to capture the collagen framework in tumor tissue by an ANN. We found that independently trained ANNs learned common aspects of tissue collagen architecture, although all models inherited expert comprehension of collagen representation via scarce, detailed, or semi-automated annotations. Trained ANNs generated sets of collagen features that outperformed conventional clinical indicators in all prognostic models obtained. In general, we show that ANNs can extract essential information embedded in bright-field pathology images and provide prognostic value in BC patients.

For the ANN to learn representations of collagen organization in a tissue image, the algorithm needs the ground truth to compute the cross-entropy loss and guide the training process. However, collagen, as an annotation object, is of a complex nature. Dekker et al.^20^ assessed breast tumor stromal organization by manually drawing straight lines along stromal fibers. To segment collagen deposition in histology images, Jung et al.^21^ generated annotations for ANN semi-automatically by image thresholding and subsequent manual refinement. In our study, we adopted annotations similar to Jung et al.^21^ to train the M3 ANN model. We also expanded the approach by Dekker et al.^20^ to train M1 and M2 ANNs to investigate the influence of cognitive bias on collagen perception by a human expert and found that human visual perception of tissue collagen framework is highly subjective, as experts’ annotations did differ significantly by all aspects evaluated (see Supplementary Table S1). Interestingly, we observed that ANNs inherit the experts’ level of detail. For example, the means of 10 of 13 Haralick texture features from M2 were significantly different between tumor grade groups, but only two from M3, and none from M1 differed significantly (see Supplementary Table S3). M2, which trained on detailed, texture-rich annotations, produced collagen framework representations that reflect tumor growth patterns by the texture features. In contrast, in M1, which was trained on scarce annotations, the tumor grade is associated with fractal dimension—a less intuitive and more complex feature that describes pattern space-filling property. In M3, tumor cell clusters disrupt dense collagen landscapes and alter the basic geometry of bulk collagen mass; thus, collagen density and straightness features define tumor shape and are associated with histological grade. Since all differently trained models independently captured aggressive tumor growth patterns by significantly emphasizing distinct collagen features (as determined by the analysis of collagen feature variance between tumor grade groups with ANOVA test having *p*-value < 0.05), it is reasonable to hypothesize that additional sources of training annotations would impact ANN performance. However, our finding that the applied annotation dilation approach allowed different models to reach a high concordance of segmentation results suggests that the proposed method effectively reacts to pronounced patterns of the collagen framework. Overall, good generalization properties of the proposed method were revealed by utilizing different annotation sources and an annotation dilation approach; thus, we expect that adding more diverse annotations would only increase the robustness of the proposed method.

Multiple studies based on SHG quantify local and global tumor collagen arrangement linking it with patient outcome. High orientation variance, local radial alignment of collagen fibers, and increased local collagen density were associated with invasion^15^ and poor patient outcome in breast carcinoma^16,20,35–37^. Similarly, collagen alignment is suggestive of the worse prognosis in pancreatic ductal adenocarcinoma^38^. In gastric cancer, increased collagen fiber width was associated with reduced patient survival^10^. We showed that similar features extracted from bright-field data allow significant patient stratification into prognostic groups in univariate analysis. The variance of orientation magnitude (M1: HR = 2.69, *p*-value = 0.029 and M3: HR = 4.07, *p*-value = 0.002), mean fiber width (M1: HR = 14.25, *p*-value = 0.010), the variance of fiber width (M3: HR = 5.01, *p*-value = 0.011), median fiber straightness (M2: HR = 0.12, *p*-value < 0.001) and texture correlation (M2: HR = 4.54, *p*-value = 0.003), after cross-validation, serve as independent indicators in multivariate (Cox regression) analysis. It is remarkable that the collagen framework data were extracted from 1 mm diameter TMA spot per patient, while no conventional clinicopathologic parameters were needed for the prognostic models (see Table 4).

TACS studies revealed exceptional prognostic information embedded in the collagen framework. Our results are in line with the concept of TACS. Latent correlations characterizing collagen arrangement in our CSMs were highly concordant between ANNs. We observed four factors where the same features from all ANNs combined (Factors 3, 6, 7, 8 see Fig. 4). TACS-1 (increased collagen deposition) resembles Factor 3, where means and standard deviations of distances between collagen endpoints (a measure of density) accumulated. Similarly, TACS-2 (straightened fibers aligned to the tumor boundary) can be observed in Factor 6 (and less clearly in Factor 8), formed by lacunarity, a measure of gappiness. Finally, TACS-3 (radially aligned collagen fibers) can be assumed in Factor 7, represented by standard deviations of orientation angles.

Of note, the proposed method is easily scalable and was tested on typical whole slide tissue images. No additional or further training of the model was needed to produce CSM from whole slide image of H&E stained tissue (see Suplementray Fig. S7).

This study has several limitations. First, training and a comparative analysis of our ANN approach based on a collagen-specific imaging technique would enable more accurate collagen annotations as used in the recent reports^22^. In particular, polychromatic polarized light microscopy^39^ that is independent of specimen placement on the slide, yet encodes the orientation into the natural-color image, could provide a promising synthesis of both methods. Second, we based our study on samples of a small amount of tumor tissue (single 1 mm TMA core per patient). Nevertheless, we still could extract significant prognostic information from the rather limited tumor tissue sample. Third, our study was limited to a small patient cohort and was designed as a post-hoc exploratory analysis. Long-term prospective studies are needed to validate our findings further.

ANN-based collagen framework image-based biomarkers can be extracted from bright-field microscopy images. Our study demonstrates that collagen framework features represented by wider collagen fibers and higher curvature, increased variance of fiber orientation magnitude, and framework texture correlation can serve as independent predictors of worse patient outcome, outperforming conventional clinicopathologic parameters used in this study.

## Supplementary Information

Supplementary Information.

## Data Availability

The datasets are available from corresponding author upon reasonable request.
