# Supplementary material for: Tumor collagen framework from bright-field histology images predicts overall survival of breast carcinoma patients
Source: Sci Rep. 2021 Jul 29;11:15474. doi: 10.1038/s41598-021-94862-6 (PMC8322324; doi:10.1038/s41598-021-94862-6)
Supplement: Supplementary file 1 — Supplementary Information. [file 41598_2021_94862_MOESM1_ESM.docx]

**Tumor Collagen Framework from Bright-Field Histology Images Predicts Overall Survival of Breast Carcinoma Patients**

Mindaugas Morkunas^1,2*^, Dovile Zilenaite^2,3^, Aida Laurinaviciene^2,3^, Povilas Treigys^1^, Arvydas Laurinavicius^2,3^

^*^ Correspondence: mindaugas.morkunas@vpc.lt

^1^ Institute of Data Science and Digital Technologies, Vilnius University, Akademijos str. 4, 08412, Vilnius, Lithuania.

^2^ National Center of Pathology, Affiliate of Vilnius University Hospital Santaros Klinikos, P.Baublio str. 5, Vilnius, Lithuania.

^3^ Department of Pathology, Forensic Medicine and Pharmacology, Faculty of Medicine, Institute of Biomedical Sciences, Vilnius University, M. K. Ciurlionio str. 21/27, 03101, Vilnius, Lithuania.

**Supplementary Figures**

Original

A1

A2

A3

Overlay


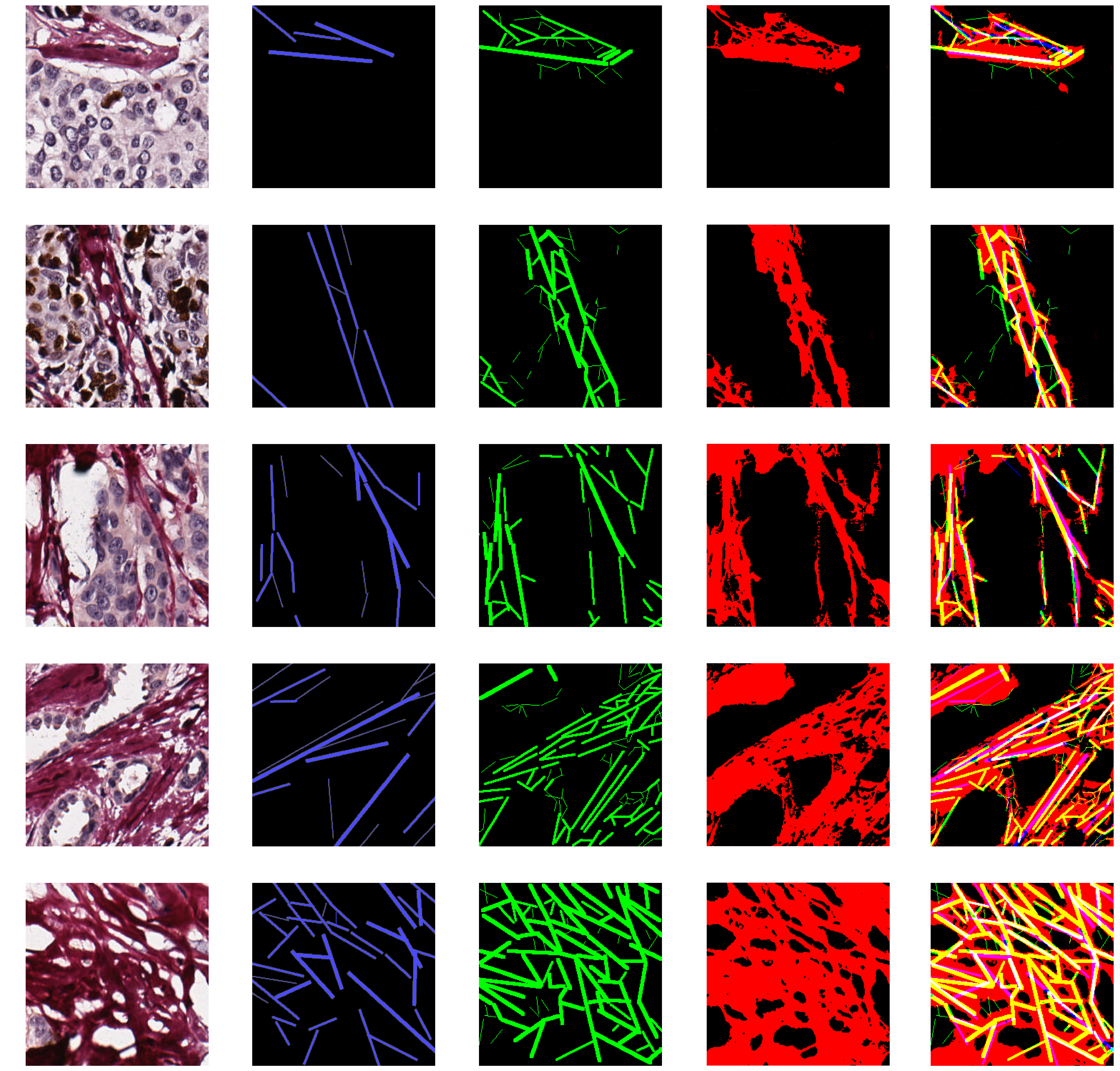


**Supplementary Fig. S1. Examples of image annotation by different methods.** The leftmost column contains 256 × 256 sized image patches of SR-stained breast carcinoma tissue, followed by binary annotation masks - low detail manual (A1), high detail manual (A2), semi-automated (A3).. Annotations are colorized for better appreciation. The rightmost column contains combined annotations to provide a visual impression of agreement.





**Supplementary Fig. S2.** Detailed architecture of an ANN. The network is a modified U-net architecture composed of 58 multichannel convolutional layers (with 3 × 3 filters and relu activations), 23 dropout layers (set to randomly ignore 20% of outputs), 5 max-pooling layers (with 2 × 2 filters), 5 transposed convolution layers (with 2 × 2 filters), and a single-channeled convolutional layer (with 1 × 1 filters and sigmoid activation) for output. The first “bottle-neck” block on the encoder path is framed by blue-dotted line.


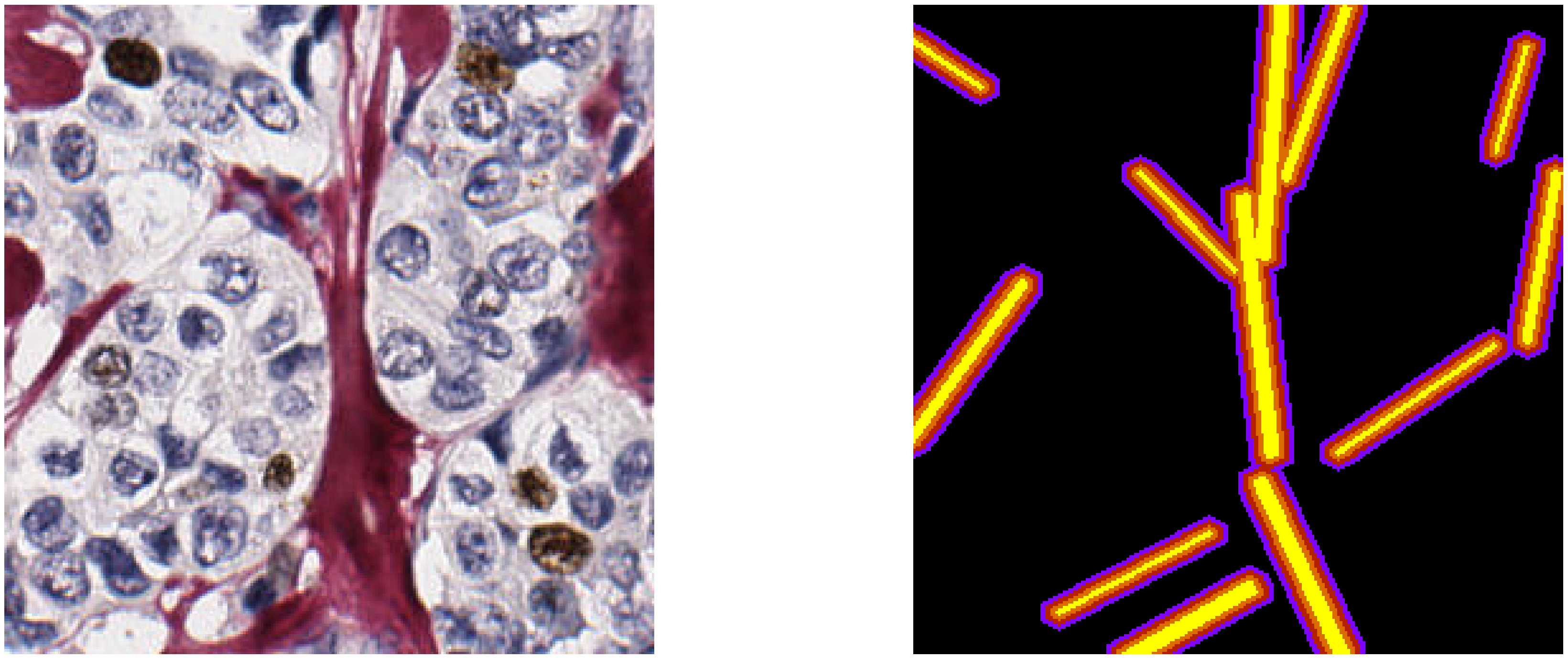


**Supplementary Fig. S3.** Annotation dilation. We applied different morphological image dilation amounts to annotation masks using a 5 × 5 elliptic structuring element and applying different number of dilation iterations (nits = 1, 2, 3). On the left, undilated annotations are colored in yellow and every additional dilation iteration gets different color.


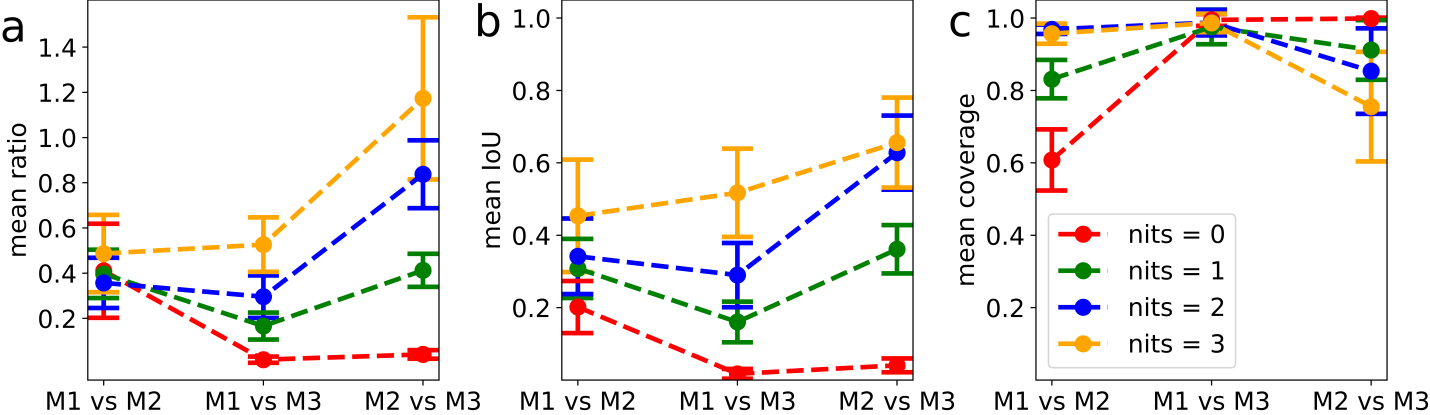


**Supplementary Fig. S4.** Segmentation consistency between trained ANNs. The agreement between CSMs from different models was measured by ratio of areas, intersection over union, and coverage. Error bars represent standard deviation, and the points are connected to better visualize the trend.


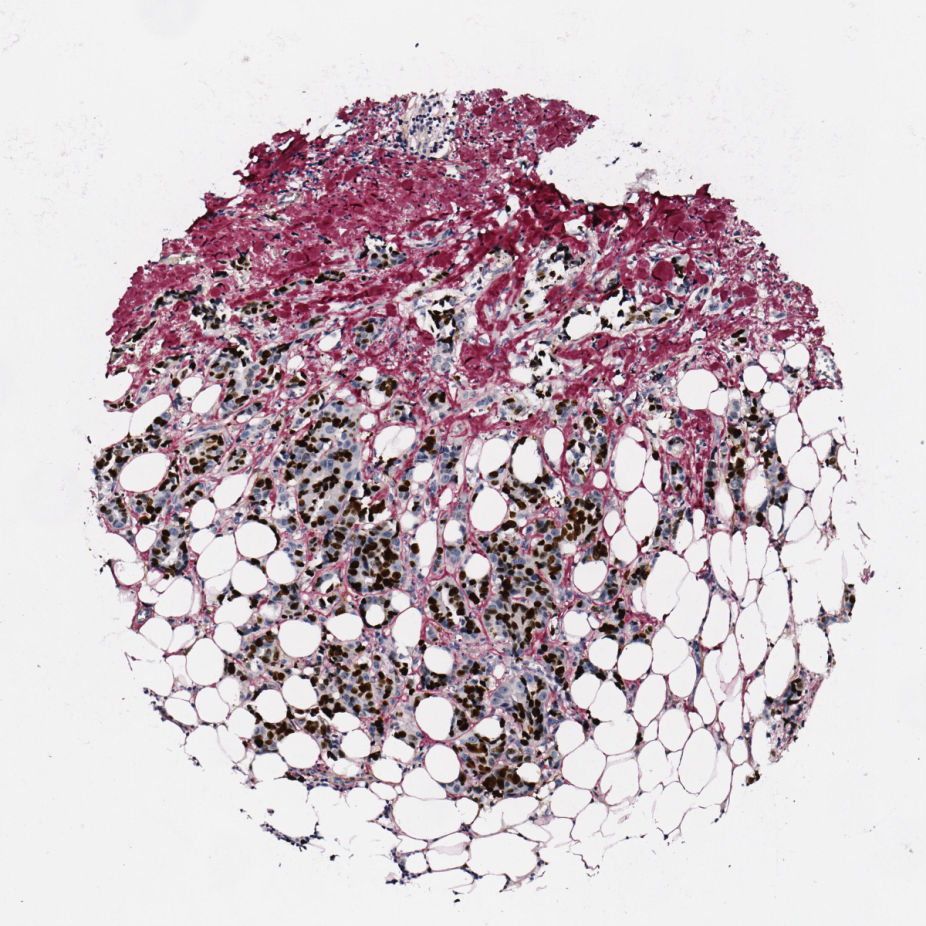

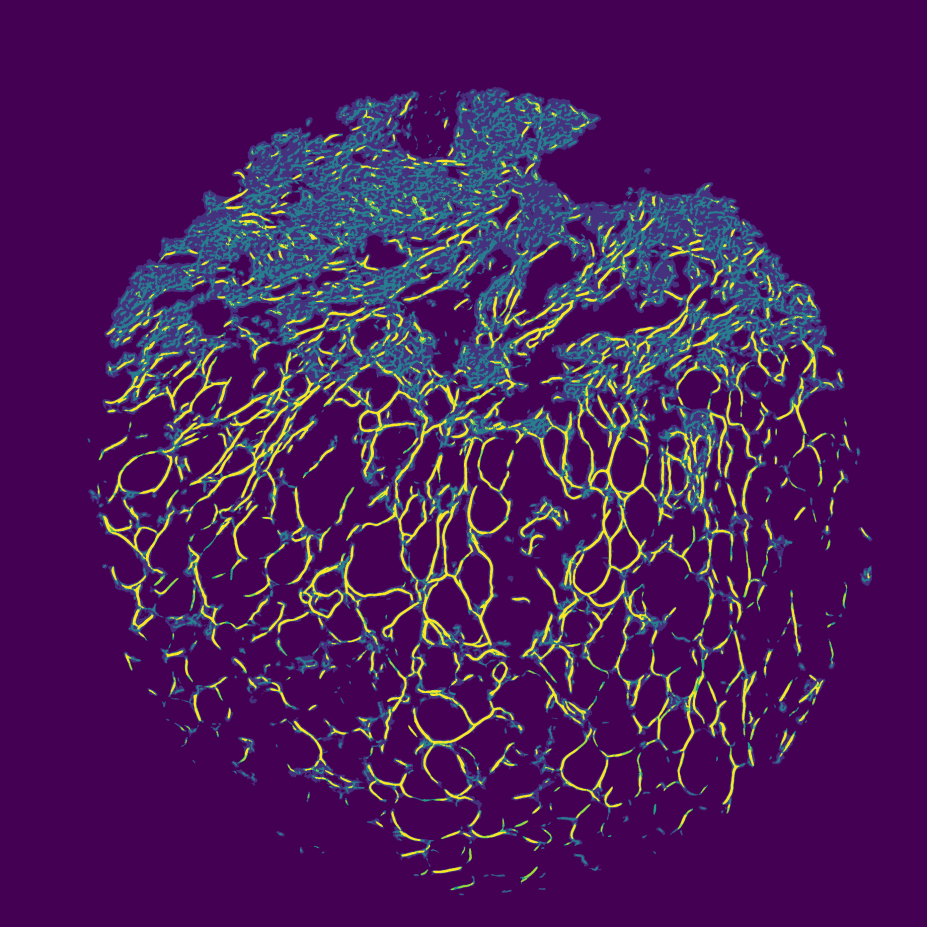


**Supplementary Fig. S5.** High-resolution examples CSMs. Original Ki67-SR-stained TMA cores (top) paired with corresponding CSMs (bottom). Overlays produced by combining CSMs from M1, M2, and M3 ANNs.


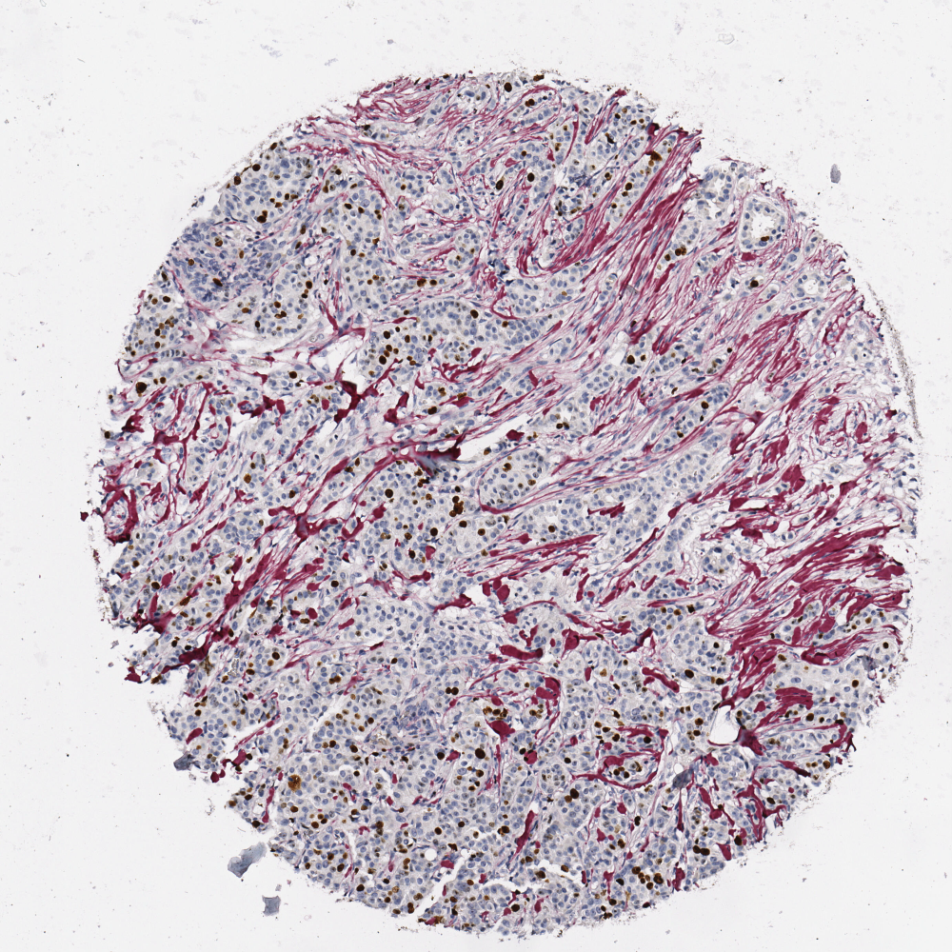

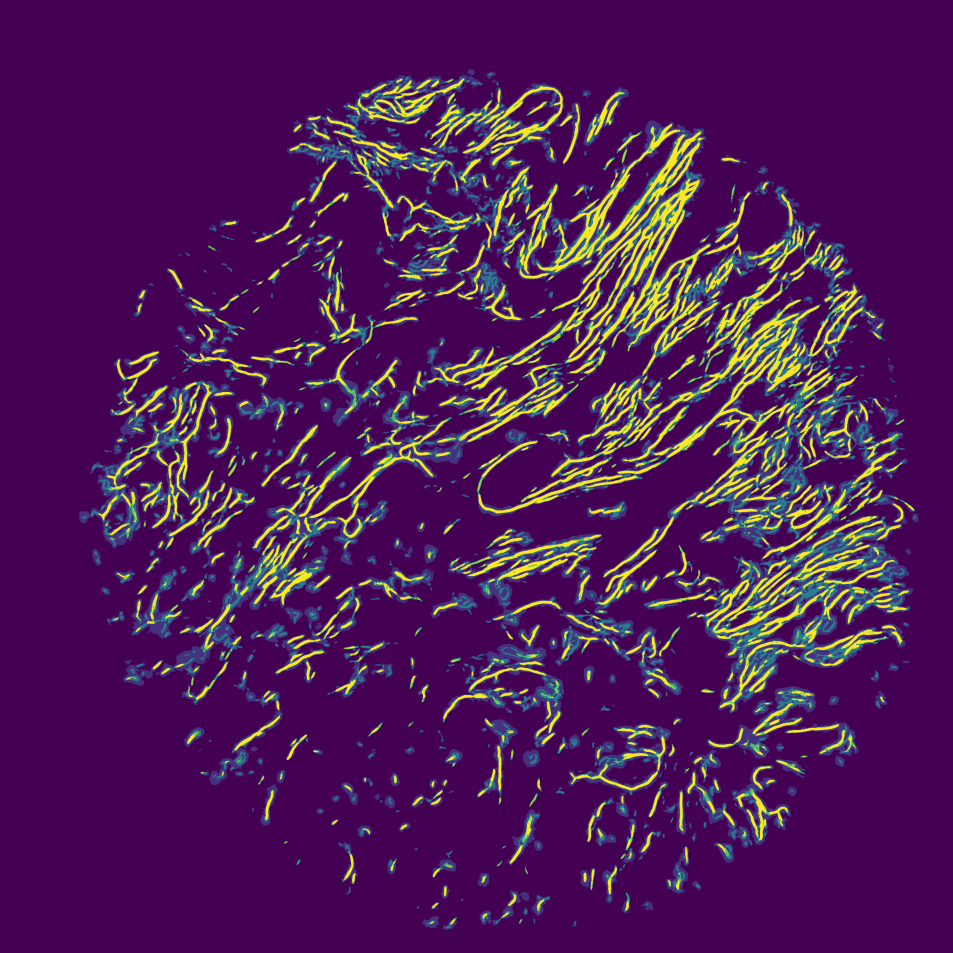


**Supplementary Fig. S5** (continued).


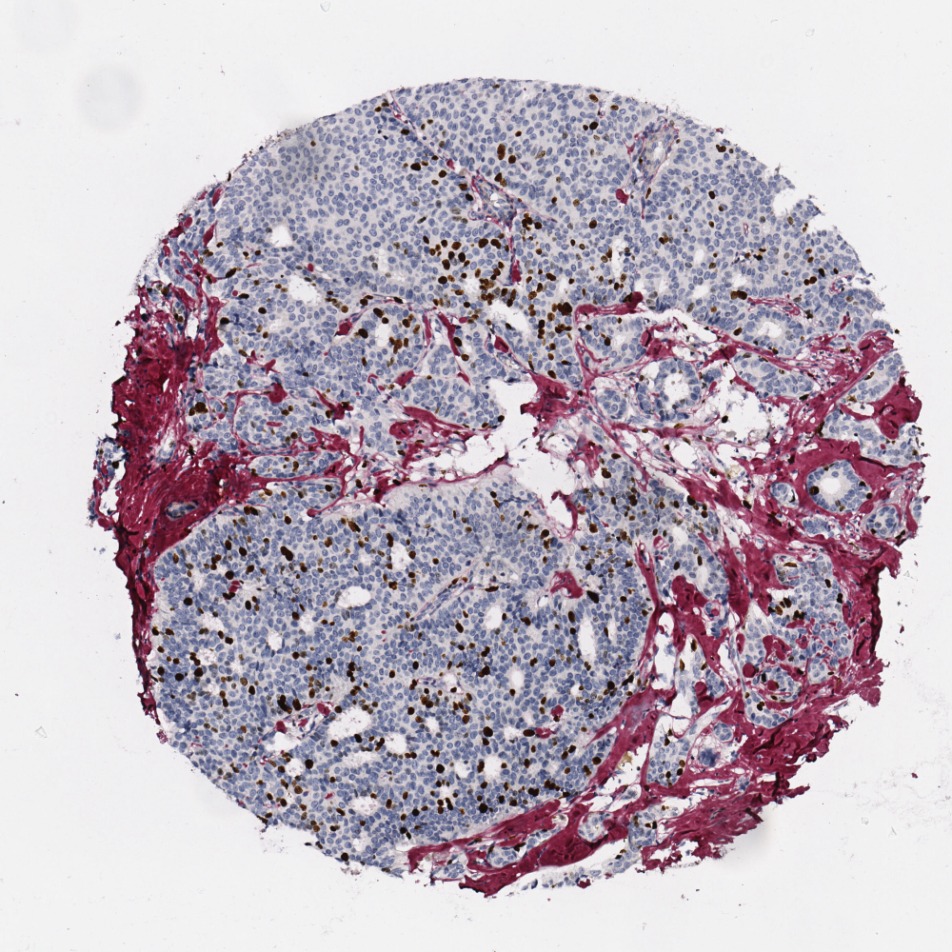

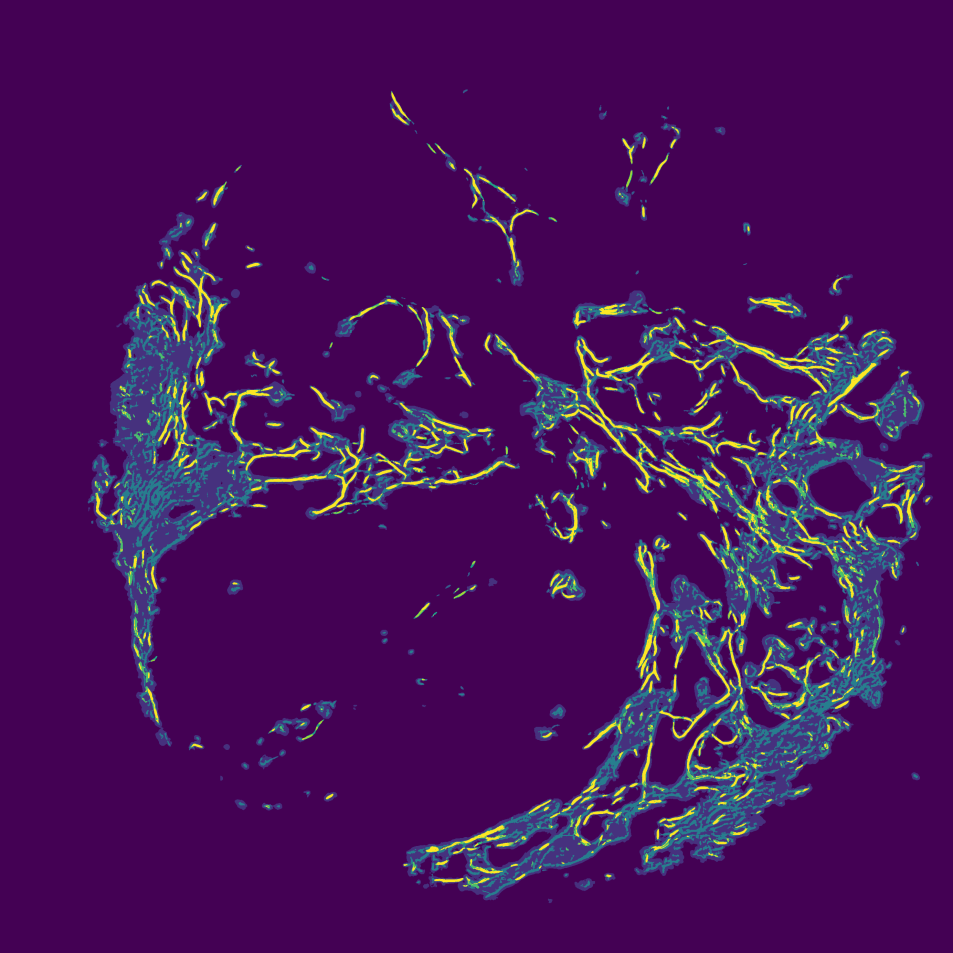


**Supplementary Fig. S5** (continued).


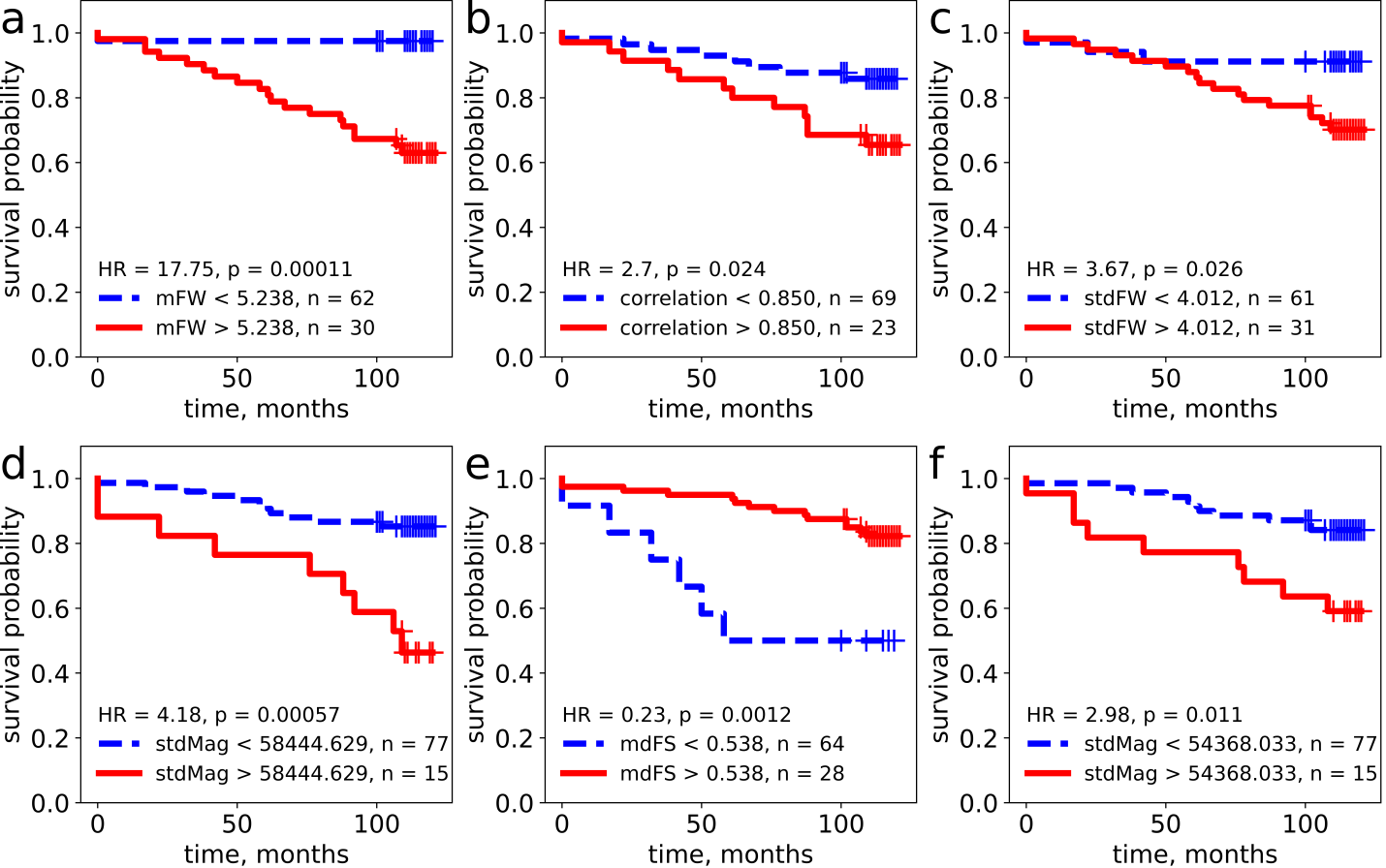


**Supplementary Fig. S6.** Kaplan-Meier survival plots with hazard ratio and log-rank test for correlation of collagen features with overall survival obtained in univariate analysis (see Table 3 in the manuscript). Mean fiber width (a) and variance of orientation magnitude (d) from M1, texture correlation (b) and median fiber straightness (e) from M2, variance of fiber width (c) and variance of orientation magnitude (f) from M3.


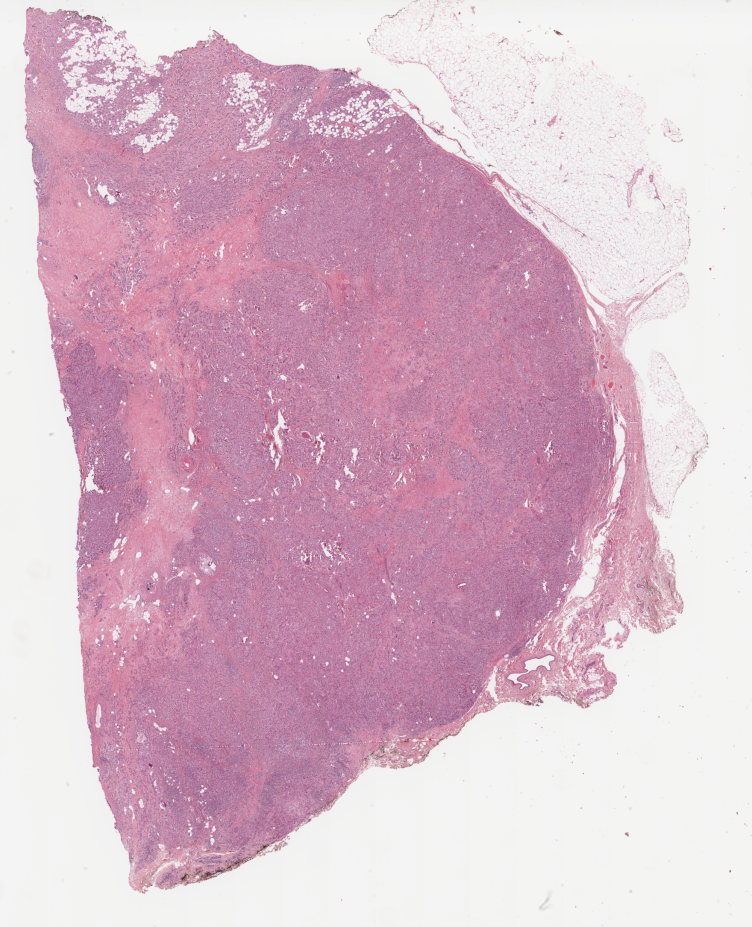

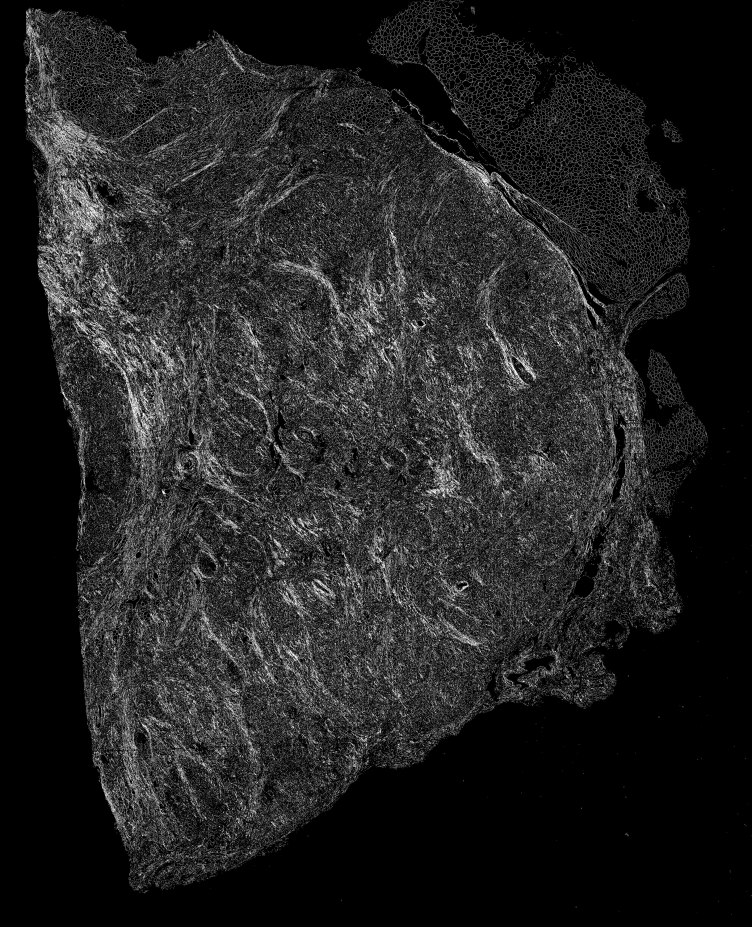


**Supplementary Fig. S7.** CSM of whole slide tissue section image. CSM example (right) was produced from H&E stained whole slide image of breast carcinoma (left) by M1 ANN model that was trained on SR-stained images exclusively. Model potentially is not limited to breast tissue type.

**Supplementary Tables**

|  | Parameters of annotated objects | | |
| --- | --- | --- | --- |
|  | Count | Size | Orientation |
| mean difference | -25.303 | 151.485 | 2.184 |
| one sample t-statistics | -13.860 | 5.718 | 2.052 |
| *p*-value | 7.914 x 10^-25^ | 1.170 x 10^-7^ | 0.043 |
| 95% limits of agreement | ± 35.423 | ± 514.076 | ± 20.652 |
| independent sample t-statistics | -13.603 | 6.897 | 0.4851 |
| *p*-value | 1.059 x 10^-29^ | 7.641 x 10^-11^ | 0.628 |

**Supplementary Table S1.** Annotation consistency between experts.

| Model | nits | Ground truth (256×256 pixel size) | Training images (256×256 pixel size) |
| --- | --- | --- | --- |
|  |  |  |  |
| M1 | 0 | 696 low detail manual annotation masks (see Supplementary Fig. S1 A1) | 696 augmented (flip, rotation) image patches (see Supplementary Fig. S1 Original) 556 patches used for training 140 patches used for validation |
|  | 1 |  |  |
|  | 2 |  |  |
|  | 3 |  |  |
| M2 | 0 | 696 high detail manual annotation masks (see Supplementary Fig. S1 A2) |  |
|  | 1 |  |  |
|  | 2 |  |  |
|  | 3 |  |  |
| M3 | 0 | 696 semi-automated annotation masks (see Supplementary Fig. S1 A3) |  |
|  | 1 |  |  |
|  | 2 |  |  |
|  | 3 |  |  |

**Supplementary Table S2.** Collagen segmentation models trained on sets of different expert-provided annotations. The amount of morphological dilation applied to the annotations is indicated by nits number (number of iterations, see Supplementary Fig.3). In total 12 model instances were trained and evaluated for segmentation consistency. Gray cells indicate models selected for further factors analysis, and univariate and multivariate prognostic analyses.

|  |  | Low grade (G1, G2) | High grade (G3) |
| --- | --- | --- | --- |
| N |  | 63 | 29 |
| M1 | *p*-value | difference (High - Low) | |
| frd | 0.027 | -0.009 | |
| M2 | *p*-value | difference (High - Low) | |
| mFS | 0.025 | -0.008 | |
| mdFS | 0.013 | -0.009 | |
| FD | 0.011 | 178782.182 | |
| nENDP | 0.005 | -3442.911 | |
| energy | 0.016 | 0.039 | |
| contrast | 0.012 | -357.176 | |
| inertia | 0.017 | -1094.767 | |
| homogeneity | 0.012 | 0.005 | |
| sum average | 0.018 | -10.439 | |
| sum variance | 0.018 | -4021.892 | |
| sum entropy | 0.014 | -0.095 | |
| entropy | 0.014 | -0.101 | |
| difference entropy | 0.012 | -0.030 | |
| mMag | 0.008 | -33410.597 | |
| stdMag | 0.040 | -15691.956 | |
| frd | 0.028 | -0.009 | |
| M3 | *p*-value | difference (High - Low) | |
| mFS | 0.004 | 0.041 | |
| mdFS | 0.001 | 0.043 | |
| FD | 0.020 | -386653.367 | |
| sum average | 0.031 | -22.110 | |
| informational measure of correlation 1 | 0.014 | 0.014 | |

**Supplementary Table S3.** Mean feature differences between tumor grade groups.

|  | Number of observations | ChiSq | Pr > ChiSq |
| --- | --- | --- | --- |
| **M1** | | | |
| mFW, stdMag | 85 | 4.749 | 0.030 |
| **M2** | | | |
| mFS, correlation, mdFS | 72 | 7.263 | 0.007 |
| **M3** | | | |
| stdFW, stdMag | 61 | 9.513 | <0.001 |

**Supplementary Table S4.** Feature selection in a leave-one-out procedure. Highest ranking set of image features for each ANN model is given.
